# Is Machine Learning a Better Way to Identify COVID-19 Patients Who Might Benefit from Hydroxychloroquine Treatment?—The IDENTIFY Trial

**DOI:** 10.3390/jcm9123834

**Published:** 2020-11-26

**Authors:** Hoyt Burdick, Carson Lam, Samson Mataraso, Anna Siefkas, Gregory Braden, R. Phillip Dellinger, Andrea McCoy, Jean-Louis Vincent, Abigail Green-Saxena, Gina Barnes, Jana Hoffman, Jacob Calvert, Emily Pellegrini, Ritankar Das

**Affiliations:** 1 Cabell Huntington Hospital, Huntington, WV 25701, USA; Hoyt.Burdick@chhi.org; 2 Marshall University School of Medicine, Huntington, WV 25701, USA; 3 Dascena, Inc., San Francisco, CA 94115, USA; clam@dascena.com (C.L.); samson@dascena.com (S.M.); abigail@dascena.com (A.G.-S.); gbarnes@dascena.com (G.B.); jana@dascena.com (J.H.); jake@dascena.com (J.C.); emilypellegrini@dascena.com (E.P.); ritankar@dascena.com (R.D.); 4 Kidney Care and Transplant Associates of New England, Springfield, MA 01104, USA; Gregory.Braden@baystatehealth.org; 5 Division of Critical Care Medicine, Cooper University Hospital/Cooper Medical School of Rowan University, Camden, NJ 08103, USA; Dellinger-Phil@cooperhealth.edu; 6 Cape Regional Medical Center, Cape May Court House, NJ 08210, USA; amccoy@caperegional.com; 7 Department of Intensive Care, Erasme University Hospital, Université Libre de Bruxelles, 1050 Brussels, Belgium; jlvincen@ulb.ac.be

**Keywords:** machine learning, COVID-19, SARS-Cov-2, hydroxychloroquine, mortality, prediction, drug treatment

## Abstract

Therapeutic agents for the novel coronavirus disease 2019 (COVID-19) have been proposed, but evidence supporting their use is limited. A machine learning algorithm was developed in order to identify a subpopulation of COVID-19 patients for whom hydroxychloroquine was associated with improved survival; this population might be relevant for study in a clinical trial. A pragmatic trial was conducted at six United States hospitals. We enrolled COVID-19 patients that were admitted between 10 March and 4 June 2020. Treatment was not randomized. The study endpoint was mortality; discharge was a competing event. Hazard ratios were obtained on the entire population, and on the subpopulation indicated by the algorithm as suitable for treatment. A total of 290 patients were enrolled. In the subpopulation that was identified by the algorithm, hydroxychloroquine was associated with a statistically significant (*p* = 0.011) increase in survival (adjusted hazard ratio 0.29, 95% confidence interval (CI) 0.11–0.75). Adjusted survival among the algorithm indicated patients was 82.6% in the treated arm and 51.2% in the arm not treated. No association between treatment and mortality was observed in the general population. A 31% increase in survival at the end of the study was observed in a population of COVID-19 patients that were identified by a machine learning algorithm as having a better outcome with hydroxychloroquine treatment. Precision medicine approaches may be useful in identifying a subpopulation of COVID-19 patients more likely to be proven to benefit from hydroxychloroquine treatment in a clinical trial.

## 1. Introduction

There are currently limited treatment options available for individuals that are infected with Severe Acute Respiratory Syndrome Coronavirus 2 (SARS-Cov-2), the etiological agent of the novel coronavirus disease 2019 (COVID-19) [1,2]. Several therapeutic agents have been evaluated in clinical trials, but robust evidence supporting their safety and efficacy is limited [3,4,5,6].

The aminoquinoline hydroxychloroquine is a well characterized medication that is used in the treatment of malaria and rheumatic diseases [7]. It has been proposed as a treatment for COVID-19 due to its anti-SARS-CoV-2 activity in vitro [8,9]. However, research examining the administration of hydroxychloroquine in the treatment of COVID-19 has not produced a clear directive for its use. Much of the initial data on the effect of hydroxychloroquine for COVID-19 were collected from studies that have either been uncontrolled or underpowered in order to identify meaningful effects on patient outcomes [5,10]. However, some of this research has indicated that adverse cardiac events, such as prolonged QT intervals and arrhythmias, have been linked to use of hydroxychloroquine in combination with azithromycin for the treatment of COVID-19 [10]. More recently, despite early evidence of benefit, several clinical trials and meta-analyses of trials have found no effect of hydroxychloroquine on COVID-19 patient outcomes [11,12,13]. A number of observational studies have reported an association between hydroxychloroquine treatment and lower mortality, which suggests a positive effect of the treatment in COVID-19 patients [14,15,16,17]. Of note, a multicenter observational study of hospitalized Italian COVID-19 patients found that the mortality reduction that was observed among patients treated with hydroxychloroquine was unlikely to be fully explained by residual confounding, as measured by the E-value [18] of 1.67 [17]. A retrospective cohort study described a similar association between reduced mortality and long-term hydroxychloroquine use in patients with rheumatic conditions [19].

As in many other trials of a new therapy, the enrolled populations have been very heterogenous and they may contain subpopulations of patients who would gain benefit or potentially harm from that therapy. Research has thus far not focused on the identification of such patients for study of potential for hydroxychloroquine benefit. Instead, controlled trials of hydroxychloroquine use traditional inclusion and exclusion criteria for entry into the study [7,20]. However, several studies have suggested a variety of COVID-19 phenotypes, including phenotypes of more severe, rapidly progressing disease that is associated with higher rates of mortality [16,21], and hyperinflammatory phenotypes that are associated with organ damage outside the respiratory system [21]. These phenotypes may have important implications for treatment effectiveness. Indeed, pharmacokinetic models have suggested that patient weight and sex impact the metabolism of hydroxychloroquine, with important implications for effective dosing [22]. The CORIST Collaboration has also suggested that patients with elevated c-reactive protein may experience greater benefits from hydroxychloroquine [17]. It is likely that more complex patient characteristics and combinations of characteristics also influence hydroxychloroquine metabolism and efficacy.

Conditions that are unique to individual patients may either restrict or facilitate their responsiveness to certain drugs. Ongoing research in the COVID-19 therapeutic space reflects an incomplete understanding of which patients may respond well to a treatment and which patients may not. Because the efficacy of any given drug is non-homogenous across patients, there is a need for finer and more accurate stratification of patient risk and response profiles in COVID-19 therapeutic research. Since the launching of the precision medicine initiative in 2015 [23], the development of treatments that account for patient heterogeneity have largely focused on personalized cancer treatment regimens [24,25,26,27,28,29]. The rapid decrease in genetic sequencing costs has enabled big-data based identification of genetic biomarkers [29], which may identify patient subpopulations more likely to respond to certain treatments. However, the widespread adoption of electronic health records (EHRs) [30] represents an equally valuable and largely untapped source of data for use in precision medicine studies seeking to identify digital biomarkers that can be used in order to predict patient responsiveness to treatment options.

Towards the end of a precision medicine approach, this study presents a pragmatic clinical trial [31] of a machine learning algorithm for the identification of patients for whom hydroxychloroquine treatment is associated with predicted survival. This methodology may lead to better patient selection criteria for clinical trial design.

## 2. Experimental Section

### 2.1. Patient Enrollment

**Figure 1:**
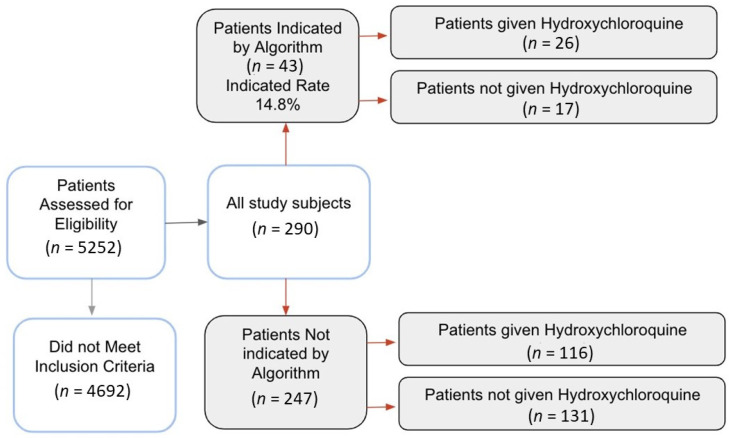
Patient inclusion flowchart.

Patients who enrolled in the IDENTIFY trial visited the emergency department or they were admitted to the hospital at six U.S. hospitals between 10 March 2020 and 4 June 2020. Patients were eligible for inclusion in the IDENTIFY clinical trial if their first set of vital sign and lab measurements were taken within 4 h of COVID-19 by polymerase chain reaction (PCR) testing and if they tested positive for COVID-19 during their visit (Figure 1); all other patients were excluded. These criteria ensured that the algorithm scores were generated for all of the patients near the time of COVID-19 diagnosis. Further details on patient inclusion criteria are presented in the Appendix A. In total, 290 patients were eligible for inclusion in our study. We enrolled all eligible patients visiting the emergency department or admitted to the hospital during the study period.

This study is considered to be of minimal risk for human subjects, as data collection was passive, and it did not pose a threat to the subjects involved. All patient data was maintained in compliance with the Health Insurance Portability and Accountability Act (HIPAA). The Pearl Institutional Review Board (IRB) approved the project was approved with a waiver of informed consent under study number 20-DASC-121, and it is registered on ClinicalTrials.gov under study number NCT04423991.

### 2.2. Data Processing

Algorithm prediction scores were generated based on three hours of patient measures. The patient scores were calculated passively and routinely every hour from the first time of available EHR measurements. For each patient, their prediction score was considered to be the algorithm score calculated closest to the time of COVID-19 diagnosis. The algorithm scores were made available to clinicians during the study period; however, there was no protocol in place requiring clinicians to access or act on algorithm scores.

The algorithm scores were computed while using diastolic blood pressure (DBP), systolic blood pressure (SBP), heart rate (HR), temperature, respiratory rate (RR), oxygen saturation (SpO_2_), white blood cell (WBC), platelet count, lactate, blood urea nitrogen (BUN), creatinine, and bilirubin. Not all data were available for all patients, and the algorithm was capable of generating scores in the presence of missing data. The machine learning algorithm was developed while using gradient boosting with XGBoost, and it was developed on independent data prior to implementation in the IDENTIFY trial.

### 2.3. Treatment

The patients were considered to be treated with hydroxychloroquine if they received it at any point during their hospitalization, before discharge or death. This study was non-interventional. Because of the non-interventional nature of the study, hydroxychloroquine doses and timing varied across clinical locations and between patients. Although physicians had access to model prediction scores, no protocol was in place for requiring that physicians access the prediction scores or utilize them in making treatment decisions.

### 2.4. Covariates

For each patient, we extracted data on potential demographic, medication, and health-related confounders. Confounders were selected based on a priori assumptions regarding relationships between covariates and on previous literature. Potential demographic factors included age and sex. Potential health-related confounders included initial oxygen saturation and past medical history, including any cardiovascular disease, history of pulmonary comorbidity (e.g., pneumonia, COPD), comorbidity that may contribute to immunocompromised state (e.g., cancer, organ transplant, diabetes, HIV), or other morbidities (including hepatic, renal, or psychiatric diagnosis). Medication use during hospitalization was also assessed, and the use of remdesivir, macrolide antibiotics, including azithromycin, angiotensin receptor blockers (ARB), angiotensin-converting-enzyme inhibitors (ACEI), and nonsteroidal anti-inflammatory drugs (NSAID) were included as a potential confounder.

### 2.5. End Point

The primary endpoint was time to in-hospital death in the algorithm indicated population. Those who were discharged alive were considered to have a competing event. The secondary endpoint was time to in-hospital death in the overall study population. The time to death was assessed in hours.

Additionally, we assessed two secondary endpoints: hospital length of stay and use of mechanical ventilation. These analyses were exploratory, and they were not adjusted for confounding factors. The average length of stay and prevalence of mechanical ventilation use was compared among hydroxychloroquine users and non-users in the general population and in the algorithm identified population.

### 2.6. Statistical Analysis

We calculated bivariate frequencies among the treated and untreated patients in order to examine associations between potential confounders and treatment with hydroxychloroquine. We also examined bivariate frequencies among patients that were identified by the algorithm to be suitable for treatment with hydroxychloroquine.

Fine and Gray models for the subdistribution hazard ratio (HR) [32] were used in order to examine the association between hydroxychloroquine treatment and time to in-hospital death, with hospital discharge treated as a competing event. This method allows for an estimation of the incidence of in-hospital death, despite the presence of a competing event that precludes the observation of in-hospital death. Incidence was estimated while using Breslow’s estimator. All of the individuals who had not experienced in-hospital mortality were censored on 4 June 2020 (the end of the study period).

We employed multivariable adjustment and inverse probability of treatment weighting (IPTW) in order to adjust for baseline confounding variables. We used logistic regression to predict the probability of treatment with hydroxychloroquine in our study population, conditional on all measured confounders, and used these predicted probabilities for constructing stabilized IPTW weights. Models for the subdistribution hazard were weighted while using IPTW with robust variance estimators, and they were additionally adjusted for age, sex, initial oxygen saturation, and presence of comorbidities. These confounders were included in both the propensity score and outcome models in order to minimize the impact of potential model misspecification in either model.

The association between treatment with hydroxychloroquine and the hazard of in-hospital death was assessed on two populations: those indicated by the algorithm as suitable for treatment with hydroxychloroquine, and the full study population. We conducted several sensitivity analyses in order to assess the robustness of our modeling assumptions. For models that were computed on both the algorithm indicated and general population, we examined subdistribution models adjusted only through IPTW, and subdistribution models only adjusted by multivariate adjustment.

Associations were visually represented through partial effects plots. Partial effects plots are a visual representation comparing the baseline survival curve of the model when the hydroxychloroquine treatment variable is varied from 0 to 1 (untreated versus treated). These plots are useful for comparing all subjects’ survival as we vary this covariate, all else being held equal. At each time point, the ratio of the values of these curves gives us the hazard ratio.

We additionally assessed adjusted hazard ratios comparing death among those that were treated and untreated with hydroxychloroquine across subgroups defined by gender, age, length of stay, initial oxygen saturation, lab measurements, and common risk scoring systems. These subgroups were examined within the whole study population (i.e., both those identified and not identified by the algorithm) with the aim of determining whether any rules-based criteria are capable of identifying patients for whom hydroxychloroquine is associated with better survival. Additionally, the feature importance of model predictors was assessed while using the Gain metric, which measures the relative contribution of each feature to the overall model. A higher Gain score implies greater importance to the model.

For all analyses, a two-sided alpha of 0.05 was used in order to determine statistical significance. All of the analyses were performed in Python version 3.6.

## 3. Results

In total, 290 patients enrolled in our study, 142 of whom received hydroxychloroquine and 43 of whom were indicated by the algorithm as more likely to have better outcomes when treated with hydroxychloroquine. Of those that are indicated by the algorithm, 26 patients received treatment with hydroxychloroquine. In the full study population, those who received hydroxychloroquine were more likely to be male and more likely to be diagnosed with acute comorbid conditions, such as pneumonia, indicating increased disease severity. Very few patients were prescribed both remdesivir and hydroxychloroquine. Table 1 displays demographic information. Table A1 presents detailed demographic information, including medical history. Differences in distribution of acute and chronic medical conditions were statistically insignificant, with the exception of initial oxygen saturation and diagnosis of sepsis.

**Figure A1:**
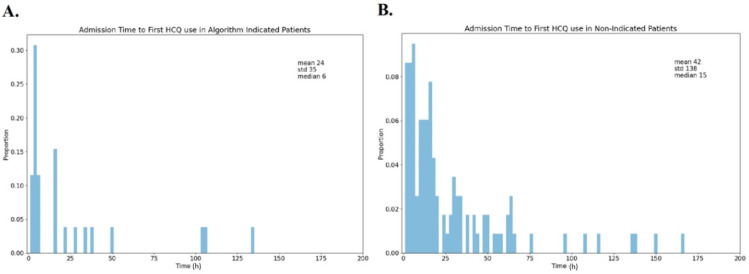
Distribution of hydroxychloroquine initiation time among (**A**) patients indicated by the machine learning algorithm and (**B**) patients not-indicated by the machine learning algorithm.

**Figure A2:**
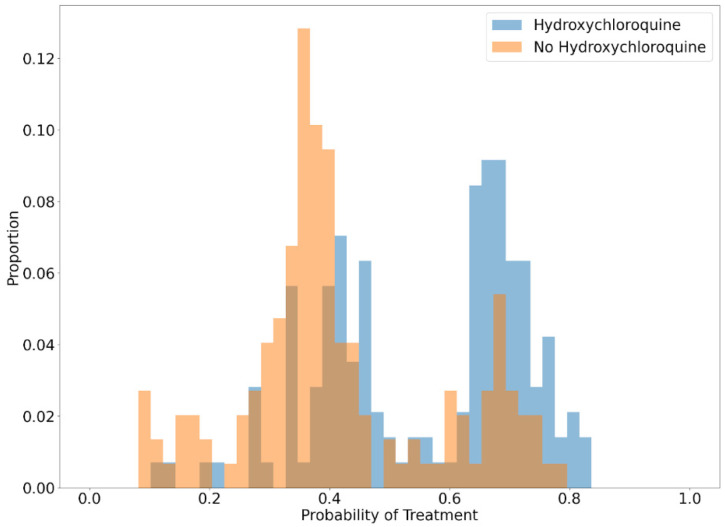
Distribution of propensity of treatment scores among those treated and not treated with hydroxychloroquine.

**Figure A3:**
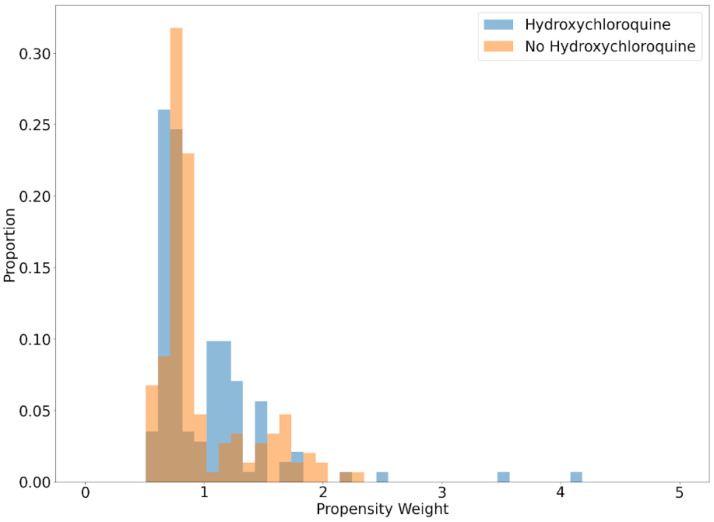
Distribution of inverse probability of treatment weighting (IPTW) weights among those treated and not treated with hydroxychloroquine.

Dosing information was incomplete in our data. However, among patients with available information on hydroxychloroquine dosing, the most common dosage was 200 mg twice a day, followed by 400 mg twice a day, each for either four or eight days consecutively. Figure A1 presents the distribution of timing of first hydroxychloroquine dose. Mean follow-up time for the full study population was 47.4 days (1138 h). Maximum follow-up time in the algorithm indicated subpopulation was 1550 h, while the maximum follow-up time in the overall population was 2200 h. In that time, a total of 63 individuals experienced the outcome of in-hospital mortality. At the conclusion of the study on June 4th, 204 patients had been discharged alive, while 23 patients remained in the hospital at the close of the study. During their hospital stay, the patients were tested for COVID-19 while using PCR at a median time of 5 h after admission. The machine learning algorithm indicated the patient to be positive for likely to benefit from hydroxychloroquine or negative for unlikely to benefit at a median time of 6 h after admission. Those that were treated with hydroxychloroquine had, on average, higher propensity scores than those not treated with hydroxychloroquine (Figure A2). No stabilized weights had a value greater than 4.2 (Figure A3).

**Figure 2:**
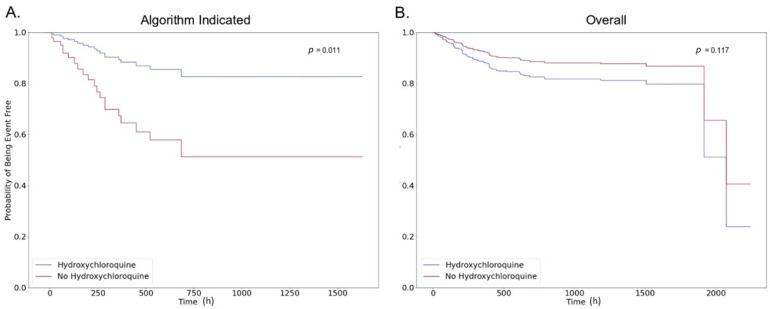
Adjusted survival curves comparing those treated and untreated with hydroxychloroquine among (**A**) those identified as suitable for treatment by the algorithm and (**B**) the full study population.

Among those that were identified by the algorithm as suitable for hydroxychloroquine treatment, hydroxychloroquine was associated with a non-statistically significant increase in survival time in the crude analysis (hazard ratio (HR) 0.53, 95% CI 0.22–1.52, *p* = 0.24). This association became statistically significant after fully adjusting for measured confounders (HR 0.29, 95% CI 0.11–0.75, *p* = 0.01). Adjusted survival among algorithm indicated patients was 82.6% in the hydroxychloroquine treated arm and 51.2% in the arm not treated with hydroxychloroquine, representing a 31.4% absolute increase in survival for the algorithm indicated patients at the end of the study period (Figure 2A).

**Figure A4:**
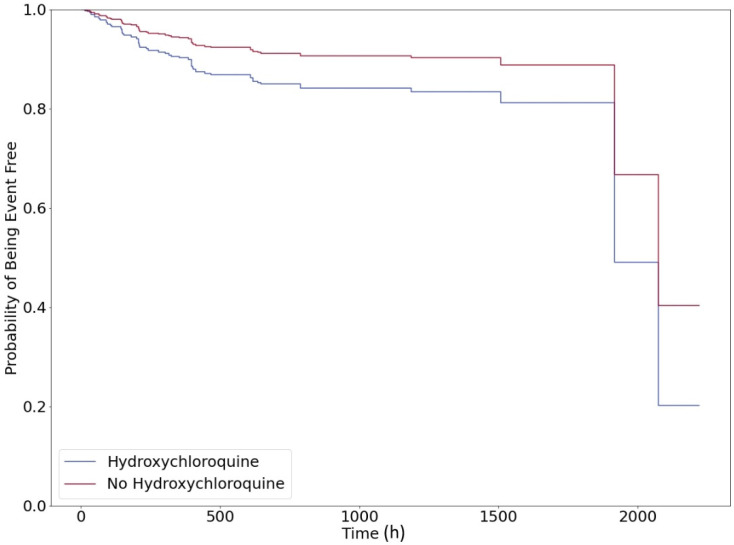
Adjusted survival curves comparing those treated and untreated with hydroxychloroquine among those identified as not suitable for treatment by the algorithm.

Among the patients not indicated for treatment by the algorithm, no benefit of treatment with hydroxychloroquine was observed (Figure A4). Similarly, in the full study population, hydroxychloroquine was not associated with increased survival in the unadjusted analysis (HR 1.20, 95% CI 0.72–1.99, *p* = 0.49). Adjustment for confounding variables supported that treatment with hydroxychloroquine was associated with a non-significant decrease in survival (HR 1.59, 95% CI 0.89–2.83, *p* = 0.12) (Figure 2B). Sensitivity analyses did not change the direction or magnitude of these associations.

Figure 2 shows the partial effects plots for patients that were identified by the algorithm and the full population, respectively. In Figure 2A, it can be seen that there is a statistically significant difference between the survival curves of patients that were identified by the algorithm who were treated with hydroxychloroquine as compared to those who are untreated. This difference is not seen across the two groups in the full population (Figure 2B). Further, we note that, in Figure 2B, the plots for the hydroxychloroquine treated and untreated groups are similar for times that are greater than 750 h. This means that the hazard ratio is close to 1 after that time period for all patients in our study, showing that there is no advantage of hydroxychloroquine for patients for whom events occur after 750 h. We also note that, for algorithm identified patients, use of hydroxychloroquine is associated with the largest impact on survival before 750 h. This means that patients with the death event happening earlier (likely indicative of more acute conditions), hydroxychloroquine treatment has a large positive impact, as reflected in the hazard ratio plots.

**Figure 3:**
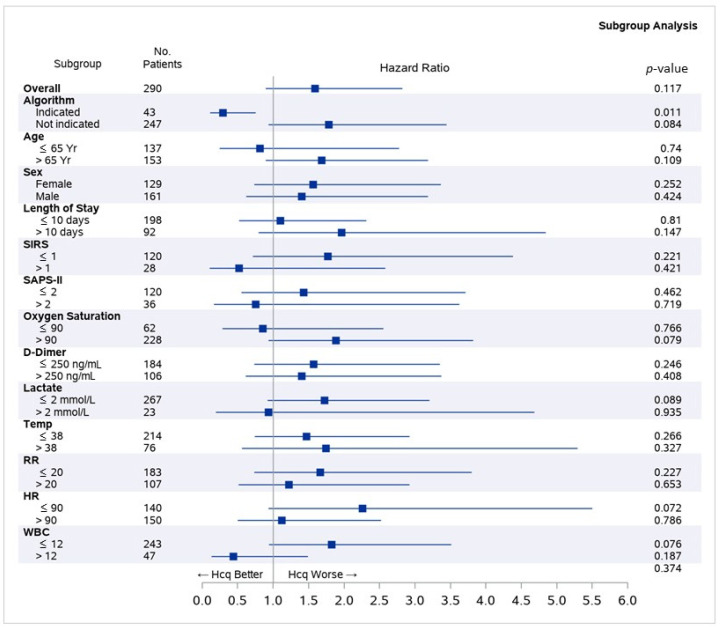
Hazard ratio of death comparing those treated and untreated with hydroxychloroquine across pre-defined subgroups. Abbreviations: HR: Heart rate. RR: Respiratory rate. SAPS: Simplified Acute Physiology Score. SIRS: Systemic Inflammatory Response Syndrome. WBC: white blood cell count.

Hazard ratios for death comparing those treated and untreated with hydroxychloroquine were statistically insignificant in all predefined subgroups, except for the one identified by the algorithm, indicating that no rules-based criteria are capable of identifying patients for whom hydroxychloroquine treatment is associated with increased survival. While several subgroups, including Systemic Inflammatory Response Syndrome (SIRS) score above 1 and Simplified Acute Physiology Score (SAPS)-II score above 2, had point estimates that indicated a potential survival benefit that is associated with hydroxychloroquine treatment, wide confidence intervals preclude making inference about the true benefit in these groups (Figure 3).

On average, those patients who were indicated by the algorithm were more likely to experience mechanical ventilation during their stay than those not indicated (Table A2). This supports that the algorithm may be identifying more critically ill patients. Among both indicated and non-indicated groups, those who were treated with hydroxychloroquine were more likely than their untreated counterparts to be ventilated during their stay. Similarly, algorithm indicated that patients had longer average hospital length of stay, again supporting greater disease severity in indicated patients. Among the algorithm indicated patients, those that were treated with hydroxychloroquine experienced longer hospital length of stay (Table A2). This may be due to fewer deaths early in hospitalization in treated as compared to untreated patients. This length of stay difference was less pronounced in the group not indicated by the algorithm.

**Figure A5:**
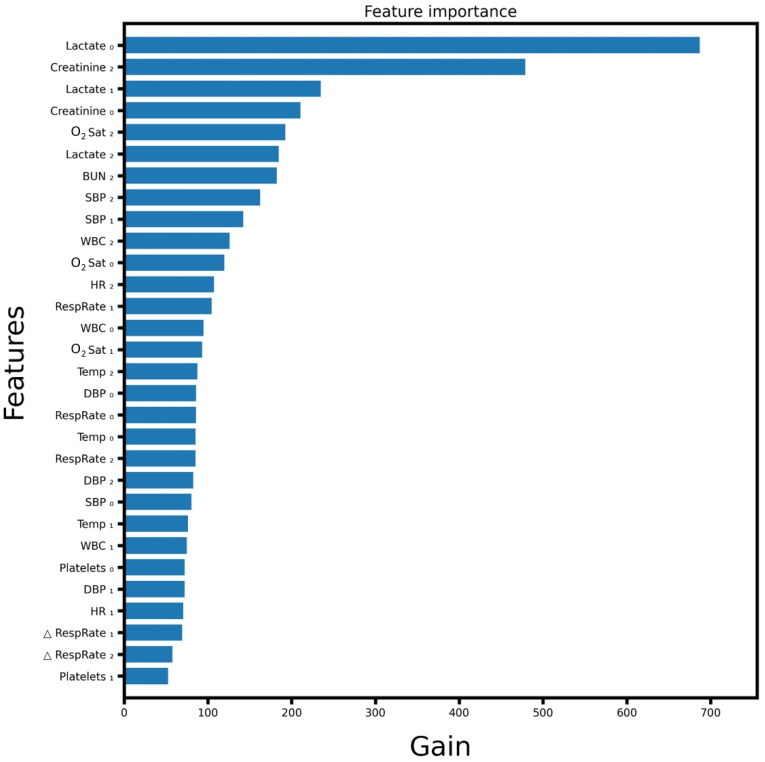
Feature Importance of algorithm inputs. Longitudinal data are used in the algorithm and thus the subscript indicates the time from which the feature was derived. For example, Lactate0 is the lactate measurement from the time at which the algorithm is applied, Lactate1 is the lactate measurement from the hour before the algorithm is applied, and so on. Δ denotes change from the previous hour of measurement. For example, Δ RespRate0 is the change in respiratory rate from the hour before the algorithm is applied to the time the algorithm is applied. Abbreviations used: BUN: blood urea nitrogen. DBP: diastolic blood pressure. HR: heart rate. O_2_ Sat: Oxygen Saturation. RespRate: Respiratory Rate. SBP: systolic blood pressure. Temp: temperature. WBC: white blood cell.

In assessing the features that are associated with model performance, lactate and creatinine at and before the time of model predictions were found to be the most important features in the patient identification algorithm (Figure A5).

## 4. Discussion

The IDENTIFY trial is the first clinical trial of a machine learning algorithm that identifies patients for whom a therapeutic intervention is associated with predicted survival in COVID-19. This study contributes to the growing body of research evaluating the effect of therapeutic agents on COVID-19 patient outcomes and it provides for more accurate stratification of patient risk and response profiles than is currently afforded in existing COVID-19 drug trials. In this study, we identified a subset of approximately 15% of the overall COVID-19 population who were predicted to have better outcomes when treated with hydroxychloroquine.

When compared to the overall population, the algorithm predicted better outcomes in those who were, on average, younger, were male, had lower initial oxygen saturation concurrent with pneumonia, and who demonstrated increased systemic inflammatory response. Uncertainty remains regarding the mechanism by which hydroxychloroquine may improve COVID-19 patient outcomes; anti-viral and anti-inflammatory mechanisms have both been proposed [33,34]. Significant evidence has been found for the proposed anti-inflammation mechanism, specifically through the inhibition of cytokine production, reducing Toll-like receptor signaling and reducing CD154 expression in T-cells [34,35], leading to the inhibition of interleukin-6, tumor necrosis factors, and interleukin-1 production. Several studies have suggested that the low dose hydroxychloroquine given early in disease course prevents mortality and intensive care unit admission; researchers have proposed that this finding is due to early anti-inflammatory treatment preventing downstream effects of inflammatory responses [35]. Similarly, the CORIST collaboration [17] found that hydroxychloroquine may be particularly effective in patients with elevated CRP levels. Consistent with an anti-inflammation mechanism of hydroxychloroquine, the algorithm’s most important inputs include markers of distributive shock often occurring from systemic inflammatory response and cytokine release syndrome. These markers include systolic blood pressure, oxygen saturation, BUN, creatinine, and lactate (Figure A4), and it is consistent with algorithm identification of patients with tissue hypoperfusion or organ dysfunction from systemic inflammatory response. Reducing inflammation may ameliorate this host response to COVID-19.

The results presented in Figure 2 demonstrate that increased survival was observed in a subpopulation of hydroxychloroquine treated patients that were identified by the algorithm. In a subpopulation of patients that were identified by the algorithm as suitable for hydroxychloroquine treatment, hydroxychloroquine was associated with 31.4% absolute increase in survival at the end of the study period in the adjusted analysis and a statistically significant hazard ratio (HR 0.29, 95% CI 0.11–0.75, *p* = 0.01). However, in the full study population, hydroxychloroquine was not associated with increased survival (adjusted HR 1.59, 95% CI 0.89–2.83, *p* = 0.12). These results support that, within the subpopulation of patients indicated by the algorithm as having better outcomes with hydroxychloroquine treatment, hydroxychloroquine was associated with a clinically meaningful improvement in survival.

Initial evidence supporting the use of hydroxychloroquine is highly variable [5,10,11]. For example, while one meta-analysis has indicated that hydroxychloroquine use appears to be safe and it may reduce the radiological progression of COVID-19 [12], another found an association between hydroxychloroquine use and increased mortality [13]. Some of the observed variability may be due to a lack of critically ill patients in many trials, small sample sizes, lack of control arms, and inclusion of concomitant antivirals in existing studies, as well as continued gaps in our knowledge regarding COVID-19 progression and variability [33]. Concerns about residual confounding make the interpretation of results difficult, even in larger observational studies that have found a decreased risk of mortality [15] or ICU admission [36] associated with hydroxychloroquine. In order to combat these weaknesses, several large randomized controlled trials (RCTs) of hydroxychloroquine for COVID-19 have been conducted. In the US, the National Institutes of Health (NIH) announced recruitment for a robust clinical trial for hydroxychloroquine to be used in conjunction with the antibiotic azithromycin [37], although recruitment has since been stopped due to insufficient enrollment [38]. The UK based RECOVERY trial found no survival benefit that was associated with use of high-dose hydroxychloroquine among COVID-19 patients [11], and a second NIH funded trial of hydroxychloroquine alone was halted when no evidence of benefit was found [39]. Several other studies [40,41,42] have found that, while hydroxychloroquine does not appear to increase the risk of harm, hydroxychloroquine does not appear to provide a survival benefit in the COVID-19 population. However, British regulators have approved continued enrollment in the COPCOV trial investigating hydroxychloroquine for the prevention of COVID-19. COPCOV enrollment had previously been paused following the null findings of the RECOVERY trial [43]. Other studies have found no effect of hydroxychloroquine for preventing infection when used as postexposure prophylaxis [19,44]. Many of these studies, including the RECOVERY trial and the World Health Organization (WHO) funded SOLIDARITY trial [45], examined the effect of only high-dose hydroxychloroquine, and the treatment timing varied across studies. For example, the RECOVERY trial administered hydroxychloroquine treatment an average of nine days after diagnosis. Recent evidence has suggested a survival benefit from lower hydroxychloroquine doses [14,17], as well as fewer side effects. The dosing of hydroxychloroquine treatment reported in earlier studies as compared to more recent studies may help to explain the variability in results, as well as the evidence of harm from hydroxychloroquine found in some studies.

Because of the variability in findings and uncertainty regarding the true efficacy of hydroxychloroquine for COVID-19, some researchers have cautioned that widespread use of the drug in clinical settings may be premature and harmful [46], although this recommendation is based on a lack of efficacy evidence, rather than convincing evidence against its efficacy. Other researchers have noted that studies establishing the efficacy of the treatment in COVID-19 patients are essential for promoting appropriate utilization of existing stores of hydroxychloroquine and ensuring that patients with rheumatic disease have continued access to the drug [47,48].

As our understanding of hydroxychloroquine treatment in COVID-19 continues to evolve and, because drug efficacy is variable across patients, it is a worthwhile research effort to identify subpopulations of patients who may benefit from receiving the treatment in order to improve patient outcomes. Studies have identified patient demographics, comorbidities, and biochemical biomarkers that roughly correlate with diverse physiological responses to SARS-COV-2 infection [49,50,51,52], recent work has aimed to define clinical criteria related to these variable responses, including defining a phenotype of hyperinflammatory COVID-19 [52], which may be helpful for identifying high-risk hospitalized COVID-19 patients. However, clinical trials of COVID-19 drug treatment efficacy face the challenge of adequate enrollment to appropriately account for heterogeneity in patient response to infection and treatment regimes. Additionally, it appears that responsive subgroups may be difficult to identify based on overt patient characteristics. Mahevas et al. [53] did not find that hydroxychloroquine improved patient outcomes in admitted patients who required oxygen. Our subgroup analysis found that no single patient characteristic was able to accurately predict positive hydroxychloroquine response. IDENTIFY is the first clinical drug trial that uses a machine learning algorithm to identify subpopulations of patients for whom hydroxychloroquine is associated with a favorable risk-benefit profile. Recent studies have suggested that the variable responses to hydroxychloroquine among COVID-19 patients may be due to factors, such as patient weight and sex [22]. Our work builds on these studies by examining the potential for more complicated combinations of patient characteristics to also impact hydroxychloroquine response. This work additionally builds on recent observational studies of hydroxychloroquine and COVID-19 [7,14] and it contributes to the larger global need for precision medicine approaches to the clinical treatment of COVID-19.

There is evidence regarding the role of machine learning as clinical decision support to guide medical treatment directions. However, these studies are largely confined to domains outside epidemiology and pharmacology [54], and more work is needed in order to examine precision medicine approaches to COVID-19 therapeutic treatments. Although an initial study by Gautret et al. reported an effective reduction of viral burden in treated patients [5], subsequent work has not upheld this finding [10]. Among the recent observational studies on hydroxychloroquine, Rosenberg et al. did not find a significant association between hydroxychloroquine treatment and differences in in-hospital mortality [20]. However, the authors noted that the observational design of the study limits the interpretation of their findings. The authors also noted that the patients who received hydroxychloroquine treatment were more likely to possess certain traits, such as having pre-existing medical conditions and impaired respiratory or liver function [20]. Similarly, Geleris et al. did not find a significant association between hydroxychloroquine administration and changes in the risk of intubation or death, but also noted that the observational design of the study limits resulting interpretations regarding the benefit or harm of hydroxychloroquine treatment [7].

This study has several limitations. While the model that is described in this study may offer an improved approach to identifying patient populations who may benefit from hydroxychloroquine treatment and while the model performs favorably in the context of recent COVID-19 work, we note that the subdistribution hazard does not have a clear causal interpretation [55]. Consequently, these findings on their own do not necessarily support a causal relationship between hydroxychloroquine treatment and direct survival benefit. A survival benefit was observed in a population of COVID-19 patients identified by the algorithm as being likely responders to hydroxychloroquine treatment, but we cannot determine, from the results of this study, what impact hydroxychloroquine may have on survival in general or on populations of patients who were not identified by the algorithm as being likely responders to the treatment. We were unable to explore the potential biological mechanisms for the survival differences found in our study. Future work comparing biological data, such as RNA titers between the treated and untreated groups and between algorithm identified and non-identified patients, would improve upon this limitation of our study.

The relatively small sample size of our study, as well as the small number of algorithm-indicated patients who received hydroxychloroquine, represents another limitation that may have reduced the power of our analyses or introduced selection bias. The distribution of follow-up time was uneven between groups. The algorithm indicated that subpopulation had a shorter maximum follow-up time, which may have introduced bias into the time-to-event analysis and interpretation of results. However, we believe any impact of this uneven follow-up time to be minimal, as the hazard ratios for all groups are close to 1 after 750 h. Additionally, information that was related to dosing of hydroxychloroquine treatment was incomplete in our observational data. Therefore, we were unable to assess dose-response relationships or control for confounding by dose of treatment. Finally, we note that, as in all non-randomized research, unmeasured confounders and multiple hypothesis testing bias may pose a threat to the validity of these results.

Further work confirming the findings of this study could include a validation cohort from a larger observational database. The characteristics of the machine learning population could also be adapted for enrollment in a standard clinical trial or for a clinical trial that randomizes subpopulations that are identified by electronic data analysis. The machine learning algorithm that is presented in this study could also be used to perform an adaptive clinical trial. In an adaptive trial design, the machine learning algorithm would identify those patient subgroups that are most likely to show no benefit from an intervention or who would be harmed by an intervention; these subgroups would then be dropped from the randomization scheme. Such studies could enrich COVID-19 therapeutics trials with positive responders, improve safety by enrolling those with a favorable risk-benefit profile, and improve patient outcomes that are related to COVID-19.

## 5. Conclusions

A machine learning algorithm has identified a subpopulation of patients as having better outcomes with hydroxychloroquine treatment. Within this algorithm identified subpopulation, treatment with hydroxychloroquine was associated with a 31.4% absolute increase in survival at the end of the study period in the adjusted analysis. These patients represented approximately 15% of the overall COVID-19 study population, which indicated that a large subset of patients may benefit from hydroxychloroquine treatment globally. These results support that precision medicine may have important applications towards identifying a subpopulation of COVID-19 patients that warrant further study. The replication of these results in a larger, interventional randomized clinical trial will serve to confirm these findings and provide further clarification on COVID-19 treatment guidelines.

## Figures and Tables

**Table 1:** Demographic characteristics of patients. All of the characteristics reported as N (%) for dichotomous variables with the exception of initial O_2_ saturation, which was measured as a continuous variable, as is reported as mean (SD).

	Demographics	Full Study Population	Treated with HCQ	Not Treated with HCQ	Indicated for Treatment by Algorithm
Age	Age < 30	10 (3.4%)	9 (6.3%)	1 (0.7%)	4 (9.3%)
	30–39	49 (16.9%)	23 (16.2%)	26 (17.6%)	6 (14.0%)
	50–59	34 (11.7%)	21 (14.8%)	13 (8.8%)	3 (7.0%)
	60–69	63 (21.7%)	28 (19.7%)	35 (23.6%)	10 (23.3%)
	70–79	70 (24.1%)	35 (24.6%)	35 (23.6%)	11 (25.6%)
	Age > 80	64 (22.1%)	26 (18.3%)	38 (25.7%)	9 (20.9%)
Gender	Female	129 (44.5%)	59 (41.5%)	70 (47.3%)	17 (39.5%)
In Hospital Conditions	Average Initial O_2_ Sat *	93.52 (5.52)	92.96 (5.45)	94.07(5.52)	89.16(7.3)
	Sepsis ^+,^*	15 (5.2%)	10 (7.0%)	5(3.4%)	6(14.0%)
	ARDS ^+^	37 (12.8%)	21 (14.8%)	16(10.8%)	9(20.9%)
	Pneumonia ^+^	40 (13.8%)	30 (21.1%)	10(6.8%)	12(27.9%)
	AKI ^+^	26 (9.0%)	13 (9.2%)	13(8.8%)	5 (11.6%)
	Arrhythmia ^+^	1 (0.3%)	0 (0.0%)	1 (0.7%)	1(2.3%)
Medications	Remdesivir	16 (5.5%)	5 (3.5%)	11 (7.4%)	3 (7.0%)
	Macrolide	130 (44.8%)	85 (59.9%)	45 (30.4%)	22 (51.2%)
	ARB	22 (7.6%)	7 (4.9%)	15 (10.1%)	2 (4.7%)
	ACEI	26 (9.0%)	16 (11.3%)	10 (6.8%)	1 (2.3%)
	NSAID	72 (24.8%)	35 (24.6%)	37 (25.0%)	9 (20.9%)
	Hcq	142 (49.0%)	142 (100.0%)	0 (0.0%)	26 (60.5%)
	Steroids	85 (29.3%)	52 (36.6%)	33 (22.3%)	16 (37.2%)

Role of the Funding Source: No funding was provided for this study. Abbreviations: ARDS: acute respiratory distress syndrome. AKI: acute kidney injury. ARB: Angiotensin Receptor Blockers. ACEI: Angiotensin-converting enzyme inhibitors. NSAID: Non-steroidal anti-inflammatory drug. HCQ: Hydroxychloroquine. **^+^** Indicates acute in-hospital conditions identified by International Classification of Disease (ICD)-10 code during the patient hospital stay. * Denotes statistically significant difference (*p* < 0.05).

## Appendix A

Study Inclusion Criteria

- Patient was admitted to the hospital as an inpatient
- Patient tested positive for COVID 19
- Patient had electronic health record data collected within four hours of receiving a COVID-19 test

**Table A1:** Detailed demographic characteristics of patients. All characteristics reported as N (%).

	Demographics	Full Study Population	Treated with Hydroxychloroquine	Not Treated with Hydroxychloroquine	Indicated for Treatment by Algorithm
Age	Age < 30	10 (3.4%)	9 (6.3%)	1 (0.7%)	4 (9.3%)
	30–39	49 (16.9%)	23 (16.2%)	26 (17.6%)	6 (14.0%)
	50–59	34 (11.7%)	21 (14.8%)	13 (8.8%)	3 (7.0%)
	60–69	63 (21.7%)	28 (19.7%)	35 (23.6%)	10 (23.3%)
	70–79	70 (24.1%)	35 (24.6%)	35 (23.6%)	11 (25.6%)
	Age > 80	64 (22.1%)	26 (18.3%)	38 (25.7%)	9 (20.9%)
Gender	Female	129 (44.5%)	59 (41.5%)	70 (47.3%)	17 (39.5%)
Diagnoses	Initial O_2_ Sat	93.52 (5.52)	92.96 (5.45)	94.07 (5.52)	89.16 (7.3)
	Sepsis	15 (5.2%)	10 (7.0%)	5 (3.4%)	6 (14.0%)
	ARDS	37 (12.8%)	21 (14.8%)	16 (10.8%)	9 (20.9%)
	Pneumonia	40 (13.8%)	30 (21.1%)	10 (6.8%)	12 (27.9%)
	AKI	26 (9.0%)	13 (9.2%)	13 (8.8%)	5 (11.6%)
	Arrhythmia	1 (0.3%)	0 (0.0%)	1 (0.7%)	1 (2.3%)
Medications	Remdesivir	16 (5.5%)	5 (3.5%)	11 (7.4%)	3 (7.0%)
	Macrolide	130 (44.8%)	85 (59.9%)	45 (30.4%)	22 (51.2%)
	Hydroxy-chloroquine	142 (49.0%)	142 (100.0%)	0 (0.0%)	26 (60.5%)
	ARB	22 (7.6%)	7 (4.9%)	15 (10.1%)	2 (4.7%)
	ACEI	26 (9.0%)	16 (11.3%)	10 (6.8%)	1 (2.3%)
	NSAID	72 (24.8%)	35 (24.6%)	37 (25.0%)	9 (20.9%)
	Steroids	85 (29.3%)	52 (36.6%)	33 (22.3%)	16 (37.2%)
History	Cardio	41 (14.1%)	11 (7.7%)	30 (20.3%)	2 (4.7%)
	Renal	5 (1.7%)	4 (2.8%)	1 (0.7%)	0 (0.0%)
	Hepatic	5 (1.7%)	3 (2.1%)	2 (1.4%)	0 (0.0%)
	Diabetes	27 (9.3%)	9 (6.3%)	18 (12.2%)	1 (2.3%)
	Organ Transplant	1 (0.3%)	1 (0.7%)	0 (0.0%)	0 (0.0%)
	HIV	1 (0.3%)	0 (0.0%)	1 (0.7%)	0 (0.0%)
	Psych	21 (7.2%)	8 (5.6%)	13 (8.8%)	0 (0.0%)
	COPD	5 (1.7%)	2 (1.4%)	3 (2.0%)	0 (0.0%)
	Cancer	32 (11.0%)	15 (10.6%)	17 (11.5%)	1 (2.3%)
	ETOH	0 (0.0%)	0 (0.0%)	0 (0.0%)	0 (0.0%)
	PNA	63 (21.7%)	31 (21.8%)	32 (21.6%)	9 (20.9%)

ARB = Angiotensin Receptor Blockers; ACEI = Angiotensin Converting Enzyme Inhibitors; NSAID = Nonsteroidal Anti-inflammatory Drug; COPD = Chronic Obstructive Pulmonary Disease; ETOH = Ethanol Alcohol; PNA = Pneumonia.

**Table A2:** Preliminary analysis of hospital length of stay and mechanical ventilation prevalence among those treated and untreated with hydroxychloroquine. Results are unadjusted for confounding factors. Hospital length of stay reported as mean (SD) in hours; mechanical ventilation reported as N (%).

	Algorithm Indicated		Not Algorithm Indicated	
	Treated	Untreated	Treated	Untreated
Hospital Length of Stay	374.6 (288.1)	147.2 (170.7)	256.2 (268.7)	229.1 (344.9)
Mechanical Ventilation	14 (53.8%)	6 (35.3%)	29 (25.0)	19 (14.5%)
